# Oxygen Saturation Imaging Using LED-Based Photoacoustic System

**DOI:** 10.3390/s21010283

**Published:** 2021-01-04

**Authors:** Rianne Bulsink, Mithun Kuniyil Ajith Singh, Marvin Xavierselvan, Srivalleesha Mallidi, Wiendelt Steenbergen, Kalloor Joseph Francis

**Affiliations:** 1 Biomedical Photonic Imaging (BMPI), Technical Medical Center, University of Twente, 7500 AE Enschede, The Netherlands; r.e.bulsink@student.utwente.nl (R.B.); w.steenbergen@utwente.nl (W.S.); 2 Research & Business Development Division, CYBERDYNE Inc., Cambridge Innovation Center, 3013 AK Rotterdam, The Netherlands; mithun_ajith@cyberdyne.jp; 3 Department of Biomedical Engineering, Science and Technology Center, Tufts University, Medford, MA 02155, USA; marvin.xavierselvan@tufts.edu (M.X.); srivalleesha.mallidi@tufts.edu (S.M.); 4 Wellman Center for Photomedicine, Massachusetts General Hospital, Harvard Medical School, Boston, MA 02114, USA

**Keywords:** oxygen saturation imaging, LED, photoacoustics, ultrasound, fluence compensation, in vivo, hypoxia

## Abstract

Oxygen saturation imaging has potential in several preclinical and clinical applications. Dual-wavelength LED array-based photoacoustic oxygen saturation imaging can be an affordable solution in this case. For the translation of this technology, there is a need to improve its accuracy and validate it against ground truth methods. We propose a fluence compensated oxygen saturation imaging method, utilizing structural information from the ultrasound image, and prior knowledge of the optical properties of the tissue with a Monte-Carlo based light propagation model for the dual-wavelength LED array configuration. We then validate the proposed method with oximeter measurements in tissue-mimicking phantoms. Further, we demonstrate in vivo imaging on small animal and a human subject. We conclude that the proposed oxygen saturation imaging can be used to image tissue at a depth of 6–8 mm in both preclinical and clinical applications.

## 1. Introduction

Blood oxygen saturation (sO ${}_{2}$ ) is the ratio of oxygen saturated hemoglobin to the total hemoglobin concentration [1]. sO ${}_{2}$ is a key physiological marker for detection, diagnosis, and treatment of several devastating diseases like cancer [1,2]. Hypoxia, reduction in sO ${}_{2}$ is one of the early biomarkers for all tumors and inflammatory diseases [3,4]. A fast-growing tumor is expected to have abnormal vasculature growth (angiogenesis) in and around it, resulting in hypoxia [5]. Detecting hypoxia at an early stage has a profound impact in early diagnosis and better treatment planning. In addition, sO ${}_{2}$ deviations inside the brain and its detection is important in the diagnosis of cerebrovascular diseases [6,7]. Hence, sensitive detection of changes in sO ${}_{2}$ is useful in early diagnosis and can help plan better treatment strategies.

In both preclinical and clinical settings, it is required to measure the changes in sO ${}_{2}$ with high spatial and temporal resolution without compromising on the imaging depth. Currently, point sO ${}_{2}$ measurement using commercial pulse oximeter is widely used to obtain an average sO ${}_{2}$ value. However, the spatial distribution of sO ${}_{2}$ is important in several applications, including tumor analysis. Non-invasive functional Magnetic Resonance Imaging (fMRI) is capable of imaging sO ${}_{2}$ changes with spatial resolutions ranging from 1–6 mm [8,9]. However, fMRI systems are too expensive and are not suitable in a point-of-care resource-limited setting. Another imaging modality that is suitable for sO ${}_{2}$ imaging is positron emission tomography (PET). The PET involves ionizing radiations and can offer far lower spatial resolution when compared to other techniques [10]. Purely optical imaging techniques can detect changes in sO ${}_{2}$ at reasonable imaging depth. However, the spatial resolution offered by optical-techniques is not sufficient for most clinical applications [11]. Photoacoustic imaging (PAI) has shown potential in multiple preclinical and clinical applications [12,13]. PAI or optoacoustic imaging is a modality that combines the advantages of ultrasound (US) and optical imaging techniques [12]. In PAI, nanosecond pulsed light excitation of optical absorbers in the tissue results in US signal generation [12]. These US signals can be detected using US transducers. Further, the signal can be used to reconstruct an image proportional to the optical absorption of the tissue. PAI is one of the fastest-growing imaging modalities of recent times, offering high-resolution images and optical contrast in deep tissue. Since acoustic detection hardware can be shared between US and PAI, it is straightforward to implement a dual-mode US and PAI system capable of offering structural, functional, and molecular contrast from a single measurement [14]. Hemoglobin is an excellent optical absorber with well-defined absorption spectra in the near-infrared wavelengths. Hence, measurement of sO ${}_{2}$ is undoubtedly the most interesting application of PAI [15,16]. By carefully selecting the light wavelengths (NIR range is commonly used because of its tissue penetration and high absorption in hemoglobin), one can measure deep-tissue sO ${}_{2}$ with unprecedented spatio-temporal resolution using PAI [17]. Conventionally, PAI utilizes bulky, slow, and expensive class IV lasers for tissue illumination [18], a key factors hindering the clinical translation of this imaging modality [19]. In recent years, there have been advancements in solid-state device technology leading to the development of laser diodes (LD) and light-emitting diodes (LED), which are suitable for PAI [20,21,22]. High power LEDs have shown potential in PAI-based superficial and sub-surface (skin and <10 mm imaging depth) imaging in both preclinical and clinical applications [23,24,25,26,27,28]. LED-PAI is portable, affordable, and energy-efficient, for that reason it may have an easy clinical acceptance. These advantages of LED-PAI will be also useful in a preclinical setting, where it is of great importance to improve small animal study outcome by monitoring disease and treatment progress [29,30]. Further, we have reported an affordable LED-based tomographic system by imaging an object from multiple angles and demonstrated it in finger joint and small animal imaging [30,31]. In this work, we propose the use of US image information for fluence compensation to improve the accuracy of sO ${}_{2}$ in dual-wavelength LED-based handheld PAI.

Fluence variations in the tissue hinder the possibility of quantitative sO ${}_{2}$ imaging [16]. It is important to develop fluence-compensation methods that are accurate, fast, and easy-to- implement in real-time PAI systems. Guo et al. utilized the acoustic spectra of PA signals to compute sO ${}_{2}$ in the tissue [32]. Deep learning-based methods were also used for sO ${}_{2}$ [33,34]. Xia et al. utilized the dynamics in sO ${}_{2}$ to correct for the fluence. Tzoumas et al. modeled the fundamental optical absorption spectra (eigenspectra) in the tissue, with the consideration of unknown optical fluence and demonstrated improvement in sO ${}_{2}$ imaging [35]. Hussain et al. used acoustically tagged photons (acousto-optics) for fluence compensation in sO ${}_{2}$ imaging [36]. All the above-mentioned works were focused on improving the quantitative nature of sO ${}_{2}$ imaging in laser-based PAI, with either algorithmic or optical methods. As most PAI systems can also perform US imaging, using US image information and the prior knowledge of tissue for fluence compensation can be a potential direction for quantitative sO ${}_{2}$ imaging in practical applications. US information was previously used for fluence estimation using Beer’s law in tissue [37]. Even though sO ${}_{2}$ imaging using LED-based PAI system was previously reported by the authors [29,38] and by Zhu et al. [25], depth-dependent fluence variation was not considered in these works. Assumption of homogeneous light distribution or simple fitting using Beer’s law is not suitable in handheld LED-PAI probes, with a gap between tissue and US probe surface caused by LED arrays placed at an angle to maximize light in the imaging plane.

In this work, we utilized the information offered by conventional US imaging to segment the tissue and used Monte-Carlo simulations of the LED probe to estimate the fluence map in the imaging plane. For the first time, we characterized the sO ${}_{2}$ imaging capability of commercially available LED-based PAI system using human blood in vitro and compared the results with oximeter readings. Further, we thoroughly tested and validated our proposed US-assisted fluence compensation method and its efficacy in improving LED-PAI based sO ${}_{2}$ imaging using tissue-mimicking phantoms with human blood at different depths and mice in vivo. Finally, we applied our proposed method in a real-time experiment showing clear differentiation between a vein and pulsating artery in a human wrist.

## 2. Materials and Methods

**Figure 1:**
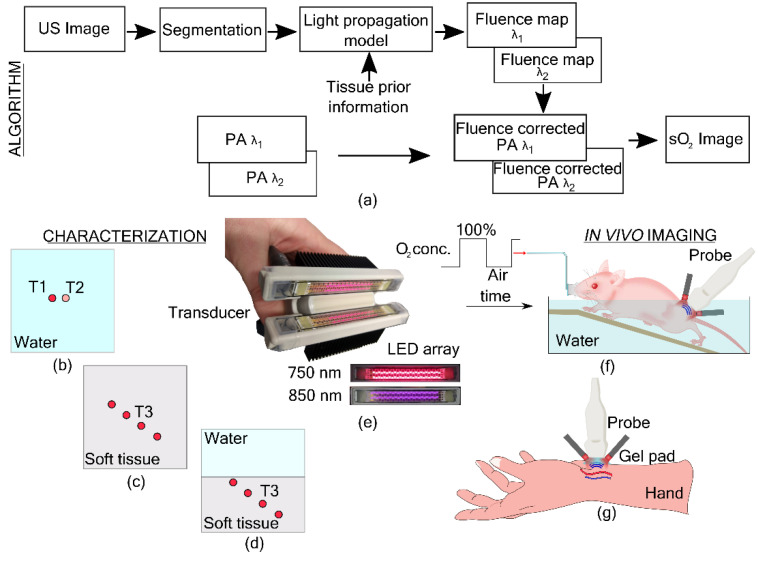
Algorithm and experimental setup. (**a**) Fluence compensation algorithm using ultrasound information. Schematic of the imaging plane in the photoacoustic oxygen saturation (PA sO ${}_{2}$ ) characterization experiments (**b**–**d**). (**b**) PA sO ${}_{2}$ imaging of tube 1 (T1) having constant sO ${}_{2}$ against T2 with varying sO ${}_{2}$ . (**c**) PA sO ${}_{2}$ imaging in soft tissue-mimicking medium with tube T3 imaged at four different depths. (**d**) PA sO ${}_{2}$ imaging in medium with two optical properties, water on the top and soft tissue-mimicking medium at the bottom. (**e**) LED-based photoacoustic probe with an ultrasound transducer and LED arrays of two wavelengths (750 and 850 nm). (**f**) In vivo imaging of a mouse with cyclically changing oxygen concentration in the breathing air. (**g**) In vivo imaging of an artery and a vein in the wrist of a human volunteer.

In this work, we used a US and LED-based PA imaging system, AcousticX (Cyberdyne Inc., Tsukuba, Japan). For oxygen saturation imaging, we used a dual-wavelength approach with an LED array having 750 nm and 850 nm elements. Figure 1e shows the probe with LED array with elements having two wavelengths. Two LED units were used in the probe on either side of the transducer (Figure 1e). The 850 nm array has pulse energy of 200 μ J and 750 nm array has 100 μ J, with a pulse duration of 70 ns. The LED array unit consists of two rows of 36 element 850 nm arrays and two rows of 24 element 750 nm arrays arranged alternately. A 128 element, 7 MHz linear US transducer with a bandwidth of 80% was used in the probe (Figure 1e). In this section, we present the proposed fluence compensation method and the details of our experimental studies.

### 2.1. Oxygen Saturation Imaging Using Linear Unmixing

An overview of PA oxygen saturation using linear unmixing with fluence compensation is presented in this section for completeness [17,39]. PA initial pressure resulting from pulsed light excitation of optical absorber with the assumption of stress confinement can be expressed as,

(1) $$p_{0}\left(r,\lambda_{i}\right)=\Gamma \mu_{a}\left(r,\lambda_{i}\right)\Phi \left(r,\lambda_{i}\right).$$

Grüneisen parameter Γ is the thermal to pressure conversion efficiency and $\mu_{a}$ is optical absorption coefficient and Φ is light fluence (integrated radiance). The optical absorption coefficient can be written as the product of the molar extinction coefficient ( ϵ ) and concentration (*c*). Thus the reconstructed PA image can be written as,

(2) $$p\left(r,\lambda_{i}\right)=\Phi \left(r,\lambda_{i}\right)\left[\epsilon_{HbR}\left(\lambda_{i}\right)c_{HbR}\left(r\right)+\epsilon_{HbO_{2}}\left(\lambda_{i}\right)c_{HbO_{2}}\left(r\right)\right]$$

Let the two wavelengths used in this study be $\lambda_{1}$ = 750 nm and $\lambda_{2}$ = 850 nm. Further, let $b(r,\lambda_{i}=p\left(r,\lambda_{i}\right)/\Phi \left(r,\lambda_{i}\right)$ be the fluence compensated PA images given by

(3) $$b=\left[\begin{array}{c} b(r,\lambda_{1})\\ b(r,\lambda_{2}) \end{array}\right].$$

The molar extinction coefficient of HbR and HbO ${}_{2}$ for both wavelengths can be combined into a matrix,

(4) $$A=\left[\begin{array}{cc} \epsilon_{HbR}\left(\lambda_{1}\right) & \epsilon_{HbO_{2}}\left(\lambda_{1}\right)\\ \epsilon_{HbR}\left(\lambda_{2}\right) & \epsilon_{HbO_{2}}\left(\lambda_{2}\right) \end{array}\right].$$

The molar concentration of HbR and HbO ${}_{2}$ ,

(5) $$x=\left[\begin{array}{c} c_{HbR}\left(r\right)\\ c_{HbO_{2}}\left(r\right) \end{array}\right],$$

can be retrieved by solving the linear equation Ax=b . The most common method to solve this linear equation is using the least square solution given by,

(6) $$x={\left(A^{T}A\right)}^{-1}A^{T}b.$$

Oxygen saturation image can then be obtained from the molar concentrations,

(7) $$sO_{2}\left(r\right)=\frac{c_{HbO_{2}}\left(r\right)}{c_{HbO_{2}}\left(r\right)+c_{HbR}\left(r\right)}\times 100\%$$

### 2.2. Fluence Compensation

Using LED arrays in the PA probe introduces some constraints. First, it is near impossible to have the transducer touching the tissue surface due to the inclined position of the LED array. Thus, a medium such as water or a gel pad is required to fill this gap for acoustic coupling. Second, with multiple LED element arrays in the unit, the light source has several mm width and cannot be assumed as a line source with both wavelengths of light originating from the same location. Further, with the large opening angle and low power of LEDs, it is not possible to assume a homogeneous illumination in the imaging plane. In this section, we present a method to estimate the fluence map inside the tissue utilizing the information from the US image. The following steps were performed to obtain a fluence compensated sO ${}_{2}$ image (Figure 1a).

1. US and PA images were reconstructed offline using a Fourier based algorithm [40]
2. The US image was segmented to obtain a binary mask of the tissue boundary.
3. The binary mask and the optical properties of the tissue were used in the light propagation model to obtain fluence maps at the imaging plane for two wavelengths.
4. PA images at two wavelengths were normalized using the fluence maps.
5. Linear unmixing was used to obtain oxygen saturation images from the fluence normalized PA images.

Characterization of the proposed approach was performed in three sets of experiments. In the first experiment, a validation of oxygen saturation imaging using the linear unmixing algorithm using the two-wavelength LED array was performed. Next, the effect of fluence compensation on oxygen saturation imaging was studied by imaging tubes carrying blood at four different depths in a soft tissue-mimicking medium. Finally, the US-assisted fluence compensation was tested on a two slab phantom and in vivo imaging.

#### 2.2.1. Ultrasound Segmentation

The tissue boundary was obtained by segmenting the US image. First, a median filter was applied to smoothen the speckles in the US image. Next, a binary image was obtained by thresholding the grayscale US image. The threshold value was manually selected for different tissue types. The binary image was then processed to obtain the tissue boundary using the Sobel edge detection operator. Further, the speckle created holes were coved using morphological operator, filling. The largest connected component was identified and used as the mask for the target tissue area. All image processing operations were performed using MATLAB (MathWorks, MA, USA) imaging processing toolbox. For the liquid phantom used in the experiment, the US information about the separating film was obtained and the water region was set to zeros.

#### 2.2.2. Light Propagation Model

A binary mask from the US segmentation and prior optical properties of the tissue was used as input to the model. We used a Monte Carlo based light propagation model (MCXLAB) for the simulations [41]. The LED-based PA probe consists of a US transducer and LED units with two-wavelength arrays. The positions of LED elements in the array, its opening angle, and pulse energy were used to define the light source. Measured optical properties of the phantom and values from the literature for the in vivo experiments at the two wavelengths were used as medium properties. The imaging plane was defined as the center slice in a three-dimensional grid and the fluence map was retrieved after the simulation from the same location.

A three dimensional geometry 55 mm (x-axis) × 55 mm (y-axis) × 37.9 mm (z-axis) ( 744×744×512 pixels) with uniform grid size of 74 μ m was used for the simulation. The acquired images (PA and US) had a dimension of 40.32 mm (x) ×37.9 mm (z) resulting from a transducer with 128 elements with a pitch of 0.315 mm and 1024 time samples at 40 MHz sampling rate. The PA and US images were then interpolated to 545×512 pixel images with a uniform spacing of 74 μ m in both dimensions. The input binary mask to the model was also the same size ( 545×512 ). The LED units have a length of 50 mm and a width of 10 mm. The LED units were placed at an angle of $41.4^{\circ }$ with respect to the transducer surface and 0.15 mm in front of it. The LED units were placed on either side of the transducer with a distance of 9.56 mm between them. Four LED element arrays, two arrays from two wavelengths (750 nm and 850 nm) are present in each LED unit. The 750 nm arrays have 24 elements and the 850 nm arrays have 36 elements. The elements were placed with a spacing of 1.4 mm within the array and the spacing between the arrays was 1.72 mm. An opening angle of 120 degree for each element was used in the model. The two wavelengths LED elements have a power ratio of 2:1 for 850 nm and 750 nm, respectively. This power ratio was incorporated in defining the light source.

The length of the input US mask (545 pixels) was smaller than the length of the LED array (744 pixels) along the x-axis. Hence, the first and the last pixels were replicated to fill the entire length of the grid, with the US mask at the center. Further, the mask is two dimensional, and to define the medium properties in the third dimension, 744 copies of the same mask were stacked along the y-axis. Two phantom experiments and two in vivo experiments were performed in this study (Figure 1). More details are provided in Section 2.3. The optical properties used in the simulation are provided in Table 1 below. The tubes used in the experiments were assumed to be transparent in the wavelengths used and hence not considered in the light propagation model.

### 2.3. *In Vitro* Characterization in Phantoms

#### 2.3.1. In Vitro Validation of Oxygen Saturation Imaging

The goal of the experiment is to check the accuracy of two-wavelength PA sO ${}_{2}$ imaging against the ground truth method, oximeter sO ${}_{2}$ . Fluence compensation was not considered in this case. However, the pulse energy of both wavelengths were used to normalize the PA images. Figure 1b shows imaging plane, where two tubes were placed in a water tank at 15 mm from the transducer surface. Both the tubes were placed at the same depth with a small distance between them so that the fluence variations are minimal. Polythene tubes (Smiths Medical, USA) with an inner diameter of 0.5 mm and an outer diameter of 0.84 mm filled with human blood were used for imaging. In the first tube (T1), blood with a constant oxygen saturation of around 65% was maintained. In the second tube (T2), different levels of blood oxygen were introduced. In this way, we also aim to see the relative difference in PA estimated sO ${}_{2}$ . The blood (9 mL with heparin as an anticoagulant) for the experiment was obtained by TechMed Centre donor services, University of Twente, from a human volunteer after completing an ethical approval, adhering to the Dutch Medical Research involving human subjects Act (WMO). One part (4 mL) of the blood was used for tube T1. To obtain deoxygenated blood, 17 mg of sodium hydrosulfite (Sigma Aldrich, Darmstadt, Germany) was added to 5 mL of blood. The deoxygenated blood was then exposed to atmospheric oxygen to obtain different levels of oxygen concentration. Blood oxygen saturation was measured in both tubes before and after the imaging using an oximeter (AVOXimeter 4000, ITC, Munich, Germany). PA images at both 750 nm and 850 nm were performed by toggling between two-wavelength arrays. PA sO ${}_{2}$ images were obtained using the linear unmixing method mentioned above. The oxygen saturation in the tube was estimated by averaging 20 PA frames, with each frame generated by averaging 64 frames on-board in the system. A region of interest of 0.6×0.6 mm was selected for each tube and the mean PA sO ${}_{2}$ value was used for the analysis. The PA estimated sO ${}_{2}$ values were then compared against the oximeter measured sO ${}_{2}$ values.

#### 2.3.2. Imaging a Homogeneous Phantom

In the second experiment, the effect of fluence compensation on sO ${}_{2}$ imaging at different depths was studied. Figure 1c shows the imaging plane where the same tube (T3) carrying blood was arranged such that it crosses the imaging plane at four different depths. An absorbing and scattering medium was used with soft tissue optical properties [42]. The optical properties of the medium are given in Table 1. Intralipid and Indian ink were used to prepare the phantom. Five liters solution was prepared in which 275 mL of 20% intralipid (Fresenius Kabi, Bad Homburg Germany) was used and 71.8 μ L Indian ink (Talens, The Netherlands) was added from a stock solution (dilution factor 69,642). Using the light propagation model, fluence maps for both the wavelengths were computed. The fluence maps were used to compensate for the non-uniform illumination in the PA images. The fluence compensated PA images were used to compute the PA sO ${}_{2}$ . PA sO ${}_{2}$ images with and without fluence compensation were compared with the measured blood oxygen saturation from the oximeter.

#### 2.3.3. Imaging a Two Slab Phantom

In a realistic scenario, the transducer cannot touch the tissue surface because of the LED arrays placed on both sides of it in an angle. In most cases, water or a gel pad is used as a coupling medium. Oxygen saturation imaging in such a scenario is studied in this experiment with a two slab phantom. Water was used for the top slab and a soft tissue-mimicking medium for the bottom slab Figure 1d. The two mediums were separated by polyurethane US film (Protection Cover Ultrasound B. V., The Netherlands) with a thickness of 140 μ m. The optical properties of the soft tissue phantom are given in Table 1. The tube arrangement used in the previous experiment at four different depths was used in this experiment as well. With the same blood in the tube, constant oxygen saturation was expected at all four depths. B-mode US imaging was performed and used to identify the two mediums. Light propagation was modeled in the two slab medium and the fluence maps were used to correct the PA images before estimating the oxygen saturation.

### 2.4. In Vivo Imaging

Two in vivo imaging experiments were performed. In the first experiment, fluence compensated oxygen saturation imaging was performed on a mouse. In the second experiment, in vivo oxygen saturation imaging was performed in the wrist of a healthy volunteer.

#### 2.4.1. Small Animal Imaging

For tracking the changes in the oxygen saturation of the blood in vivo, we conducted a small animal study. The animal (BALB/c nude mice) was anesthetized with Isoflurane vapor added to the breathing air through a nose cone (Figure 1f). The transducer as well as the lower part of the mice body were immersed in a warm water bath for acoustic coupling (Figure 1f). The flank region closer to the thigh was aligned to the imaging plane using US imaging. A combination LED array (750 nm and 850 nm) was used at a repetition rate of 4 kHz, and the resulting PA signal was averaged 640 times to display PA image with a frame rate of 6 Hz. At first, the animal was allowed to breathe in normal air ( 21% oxygen) and the oxygen saturation images were acquired. Then the breathing gas was changed to 100% oxygen and the animal was allowed to stabilize to the oxygen level for two minutes. US and PA raw RF files were saved for the entire duration. The US image was used to identify the tissue boundary. Optical properties of mouse tissue used in the light propagation model are given in Table 1. Fluence compensated oxygen saturation images were computed. The oxygen levels were compared for both levels by selecting three blood vessels as regions of interest.

#### 2.4.2. Human Imaging

In the final experiment, the wrist of a human volunteer with brown skin was imaged. An artery and vein were identified and imaged in real-time (30 Hz) using both the B-mode US and two-wavelength PA imaging. US-assisted fluence compensation was performed, and oxygen saturation images were computed. From the PA sO ${}_{2}$ image, the artery and vein regions were analyzed to estimate the mean sO ${}_{2}$ values by computing the mean over a region of interest.

## 3. Results

### 3.1. In Vitro Validation of PA sO ${}_{2}$

**Figure 2:**
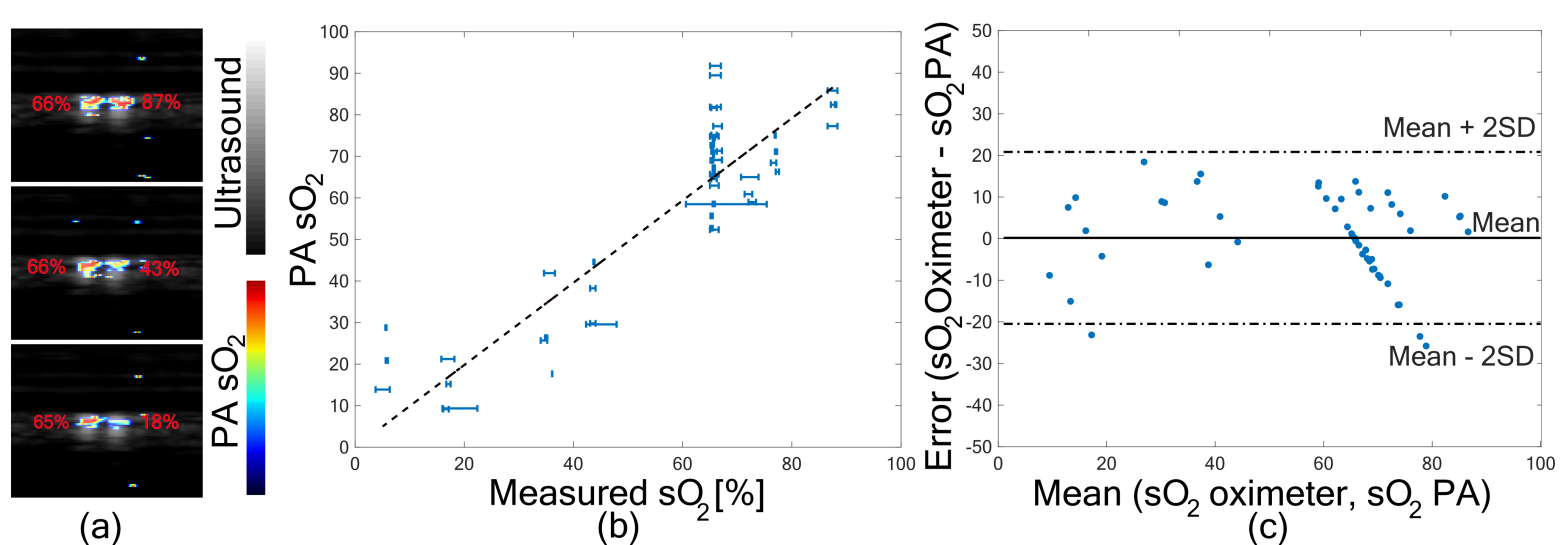
Photoacoustic Oxygen saturation (PA sO ${}_{2}$ ) validation. (**a**) PA sO ${}_{2}$ images from three blood oxygen levels with oximeter reading marked in the image. (**b**) PA sO ${}_{2}$ plotted against measured sO ${}_{2}$ from oximeter readings. (**c**) Error plot showing average sO ${}_{2}$ from oximeter values and PA sO ${}_{2}$ plotted against difference in sO ${}_{2}$ between the two methods.

Validation of PA sO ${}_{2}$ imaging is presented in this section to analyze the accuracy of the two-wavelength PA sO ${}_{2}$ against a ground truth method. Figure 2a shows two tubes having blood with three different levels of oxygen. Oximeter readings are marked next to the tubes, and the difference in oxygen saturation is visible in the PA sO ${}_{2}$ images. To quantify the changes in oxygen saturation and validate it with oximeter readings, PA sO ${}_{2}$ in a region of interest of the tubes are compared in Figure 2b. The graph shows the PA estimated sO ${}_{2}$ against the measured oxygen saturation. sO ${}_{2}$ readings before and after imaging are indicated by the error bar in the measured sO ${}_{2}$ . A linear fit to the data is shown with the dashed line. The correlation coefficient (r) between measured sO ${}_{2}$ and PA estimated sO ${}_{2}$ is 0.893 (*p* < 0.001), which shows that the two measurements are linearly correlated. The error in the PA estimated sO ${}_{2}$ , given by the difference between the two methods is plotted in Figure 2c. A mean error of 0.18% and a standard deviation of 10.33% was observed. Most of the error values are around the mean, and 94% of the values are within the Mean ± 2SD region, indicating that the variations are from the noise in the PA measurements.

### 3.2. Fluence Compensated PA sO ${}_{2}$

**Figure 3:**
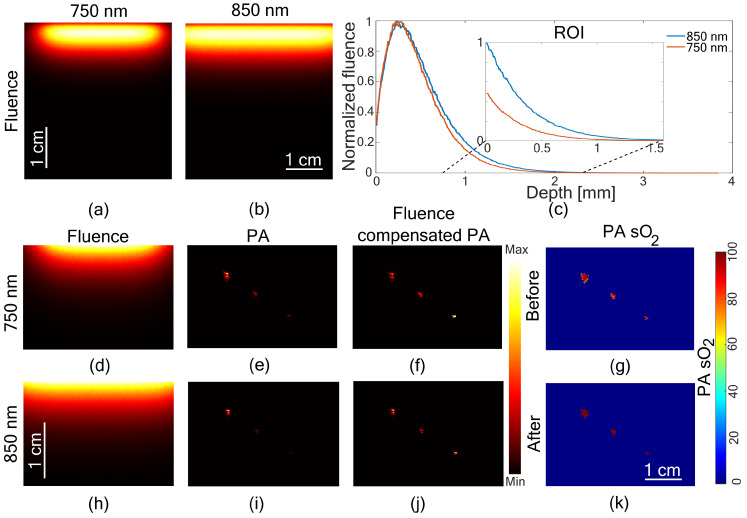
Fluence compensation in a homogeneous medium. Fluence map at the imaging plane from (**a**) 750 nm and (**b**) 850 nm. (**c**) Line profile showing normalized fluence in the depth direction and the zoomed-in region showing fluence decay in the Region Of Interest (ROI), marked with dashed lines. Fluence map in the ROI with (**d**) 750 nm and (**h**) 850 nm. Uncompensated photoacoustic (PA) images at (**e**) 750 nm and (**i**) 850 nm and corresponding fluence compensated PA images (**f**,**j**). Photoacoustic oxygen saturation images (PA sO ${}_{2}$ ) (**g**) before fluence compensation and (**k**) after fluence compensation.

In the second experiment, a homogeneous phantom was considered with soft tissue optical properties (Table 1). Fluence for both 750 and 850 nm is shown in Figure 3. The peak fluence can be observed at the LED locations in Figure 3a,b. Due to the advanced position ( 9.2 mm) of LED arrays on the sides of the transducer, this region in the imaging plane cannot be used for imaging tissue. We only considered the imaging plane below the LED position as the region of interest (ROI). Figure 3c shows a normalized line profile through the fluence map for the entire imaging plane and in the ROI. Figure 3d,h shows fluence map at 750 and 850 nm, respectively. Figure 3e,i shows the PA images of the same tube with blood at different depths at 750 and 850 nm, respectively. Figure 3f,j shows fluence compensated PA images. PA images show the signal from the tube located at three depths. With fluence compensation an enhancement in the PA signal from deeper locations is visible. Figure 3g shows the PA sO ${}_{2}$ image before fluence compensation and Figure 3k shows PA sO ${}_{2}$ image after fluence compensation.

Fluence drop of 1/*e* was observed at a depth of 3.6 mm for 750 nm and 4.2 mm for 850 nm. The maximum depth obtained in the system with the denoising filters was 10.5 mm and using the offline program it was 6 mm. The measured oximeter readings of the blood in the tube before imaging was 95.9% and after imaging it was 96.6% . The PA estimated sO ${}_{2}$ at three tube locations (mean value) before fluence compensation were 85.8%,83.9% and 81.7% and after fluence compensation were 99.1%,99.3% and 99.6% . In the offline reconstruction, the fourth location of the tube was not visible. However, all four tubes were visible in the system.

**Figure 4:**
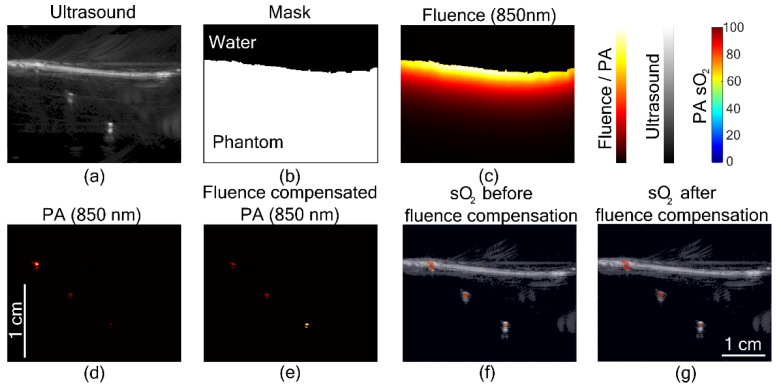
Ultrasound-assisted fluence compensation. (**a**) Ultrasound image of the two-layer phantom. (**b**) Binary mask obtained by segmenting ultrasound image. (**c**) Fluence map at 850 nm. (**d**) Photoacoustic (PA) images before fluence compensation at 850 nm and corresponding (**e**) fluence compensated image. Photoacoustic oxygen saturation (PA sO ${}_{2}$ ) images (**f**) before fluence compensation and (**g**) after fluence compensation overlaid on the ultrasound image.

In the third experiment, a two-slab phantom was considered. The upper region was water and the lower region a soft tissue-mimicking phantom. Figure 4a is the US image showing the film separating the two mediums. Figure 4b is the segmented image with ones for soft tissue region and zeros for water. Figure 4c shows the fluence in the medium at 850 nm. The PA image of the tube at three different depths is shown in Figure 4d. The fluence compensated PA image in Figure 4e shows the enhancement at deeper locations. Figure 4f shows the sO ${}_{2}$ image before fluence compensation overlaid on the US image and Figure 4g is sO ${}_{2}$ image after fluence compensation. The enhancement in the PA sO ${}_{2}$ is visible in Figure 4g. The PA sO ${}_{2}$ estimated by taking the mean value for the tube locations before fluence compensation were 82.4%,76.9% and 77.7% , and fluence compensation it was 91.8%,89.5% , and 92.1% . The oximeter readings before and after imaging were 96.8% and 97.4% , respectively.

### 3.3. In Vivo PA sO ${}_{2}$ Imaging

**Figure 5:**
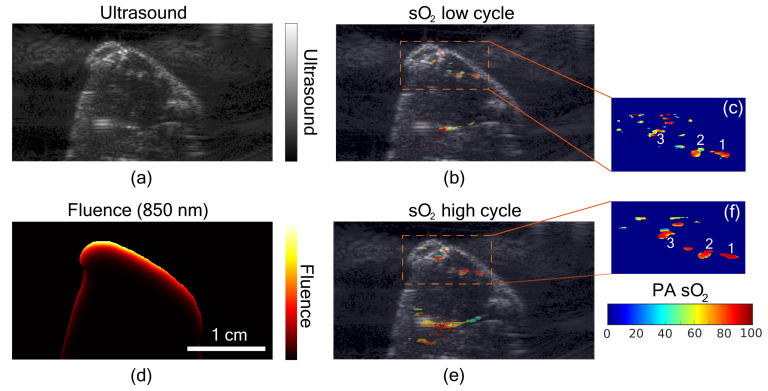
In vivo small animal imaging. (**a**) Ultrasound image of the thigh muscle of a mouse. (**b**) Fluence compensated photoacoustic oxygen saturation (PA sO ${}_{2}$ ) during the low sO ${}_{2}$ cycle overlaid on the ultrasound image. (**c**) Zoomed-in region of the PA sO ${}_{2}$ image from the low sO ${}_{2}$ cycle. (**d**) Fluence map at 850 nm obtained using segmented ultrasound image. (**e**) Fluence compensated PA sO ${}_{2}$ during the high sO ${}_{2}$ cycle, overlaid on the ultrasound image. (**f**) Zoomed-in region of the PA sO ${}_{2}$ image from the high sO ${}_{2}$ cycle.

Results from in vivo PA sO ${}_{2}$ imaging on mice thigh muscle is shown in Figure 5. The animal was given two levels of oxygen, switching between normal air and 100% oxygen. Figure 5a shows the US image of the thigh muscle of the mice. The fluence map at 850 nm utilizing the US segmented image is shown in Figure 5d. Figure 5b,e show fluence compensated PA sO ${}_{2}$ images corresponding to the normal air (low cycle) and 100% oxygen (high cycle), respectively. The zoomed-in region from both low and high cycle is shown in Figure 5c,f, respectively. The difference in sO ${}_{2}$ images is clear between normal air and 100% oxygen. To quantify the change, three regions marked by 1, 2, and 3 in Figure 5c,f were analyzed for the mean PA sO ${}_{2}$ values. During the low cycle PA sO ${}_{2}$ values were 72.9%,67.8% and 67.3% , respectively, for regions 1,2 , and 3 and it changed to 93.4%,89.2% and 85.4% during high cycle.

**Figure 6:**
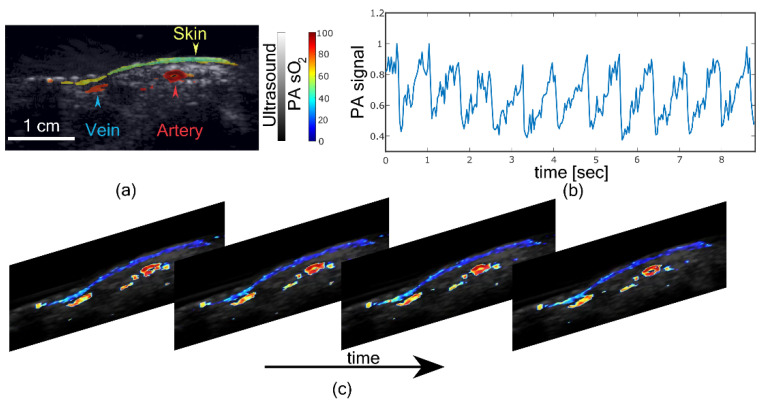
In vivo imaging on a human wrist. (**a**) Fluence compensated photoacoustic oxygen saturation (PA sO ${}_{2}$ ) image of the human wrist overlaid on an ultrasound image. (**b**) Normalized photoacoustic (PA) signal from artery showing pulsation. (**c**) PA sO ${}_{2}$ frames showing pulsating artery. (Video S1).

In the final experiment, an in vivo imaging on the wrist of a healthy volunteer was performed. Figure 6a shows fluence compensated PA sO ${}_{2}$ image overlaid on US image. An artery on the right side and a vein on the left side of the PA sO ${}_{2}$ image is visible, along with the skin signal. An artery is characterized by higher sO ${}_{2}$ while a low sO ${}_{2}$ for the vein. The estimated PA sO ${}_{2}$ by selecting a region of interest in the artery was 92.6% and that of the vein was 78.7% . The difference in the sO ${}_{2}$ between artery and vein is evident in Figure 6a. Mean PA signal acquired real-time (30 Hz) from the artery at 850 nm is presented in Figure 6b, which shows the pulsation due to the heart rate. The estimated heart rate of the subject from the PA signal was 84 beats per minute. Figure 6c shows the real-time (without fluence compensation) PA sO ${}_{2}$ images from the system. Figure 6c shows the pulsating artery, demonstrating the real-time PA sO ${}_{2}$ imaging capability of LED-based PA sO ${}_{2}$ imaging.

## 4. Discussion

In recent years, LED-PAI has shown potential in various clinical and preclinical applications. However, depth-dependent fluence variation was not considered for PA sO ${}_{2}$ calculation. An accurate fluence compensated PA sO ${}_{2}$ for LED-PAI is expected to accelerate the clinical translation of LED-PAI. In this work, we characterized the accuracy of PA sO ${}_{2}$ imaging by using in vitro human blood and compared the PA sO ${}_{2}$ with conventional oximeter readings. A good correlation of 0.893 (*p* < 0.001 ) was achieved between measured sO ${}_{2}$ and PA sO ${}_{2}$ . We utilized the structural information offered by US images for tissue segmentation. Further, the segmented tissue information was used in the light propagation model using Monte-Carlo simulations to compensate for fluence variations to improve the sO ${}_{2}$ imaging accuracy. The fluence compensation was validated using two different tissue-mimicking phantoms with human blood-filled tubes at different depths. The sO ${}_{2}$ values were compared before and after the fluence compensation step. In both phantoms, blood with the same oxygenation level was present at different depths. It is evident from our results (decreasing sO ${}_{2}$ values with depth) that it is important to account for light fluence variations to estimate sO ${}_{2}$ accurately. After applying the fluence compensation algorithm, the PA sO ${}_{2}$ imaging accuracy improved by 12% in the two slab phantom and 15% in the homogeneous phantom studies. The best enhancement was achieved for deeper locations in comparison to the uncompensated PA sO ${}_{2}$ imaging. An error of 15% (when no fluence compensation was performed) is not acceptable in a clinical scenario, especially when one has to differentiate an artery and a vein (difference in sO ${}_{2}$ for arterial and venous blood is less than 15–20%). To demonstrate the potential of fluence compensated PA sO ${}_{2}$ imaging in vivo, we performed a small animal study in which the thigh muscle of a live mouse was imaged. First, the mouse was set to breathe normal air ( 21% ) and then switched to ( 100% ) oxygen and PA sO ${}_{2}$ was monitored to see the dynamic imaging capability. Our approach yielded expected results showing differences in sO ${}_{2}$ values of different blood vessels (67–73% sO ${}_{2}$ during normal air-breathing and around 85–94% during 100% oxygen breathing) with an imaging depth of approximately 6 mm. Finally, we also performed a fluence-compensated PA sO ${}_{2}$ measurement on the wrist of a human volunteer and imaged a vein and a pulsating artery for which a difference of 14% sO ${}_{2}$ was observed.

We foresee that the implementation of this idea in the imaging system will open up the possibility of improving the sO ${}_{2}$ imaging capability. Since US imaging is already available in the system, this data can be used for tissue segmentation and consequently compute a fluence map specific for the tissue and compensate the PA data for sO ${}_{2}$ estimation. We believe that our approach is suitable for any PAI system with US imaging capability. In this work, we were able to achieve a penetration depth of around 10 mm in phantom experiments when sO ${}_{2}$ imaging was performed along with conventional US imaging in the AcousticX system. For the in vivo experiments, we achieved an imaging depth of 6 mm, which is good enough for small animal imaging and human wrist imaging that we have demonstrated. However, it is critical to improve the imaging depth to explore a wide range of clinical applications. Improving the LED array packaging, pulse repetition rate, and coded-excitation schemes may be some aspects to be considered in this direction [45]. Further, novel deep learning and image processing methods are also expected to have an impact in improving spatial resolution and imaging depth of LED-PAI [46,47,48]. In this work, we used a combination LED array of 750 nm and 850 nm with a pulse energy of around 200 μ J and 100 μ J per pulse, respectively. As one can see, pulse energy of 750 nm is just 100 μ J, which is half of that of 850 nm, and this is the main factor hindering the imaging depth in this work. The higher amount of background noise present in the 750 nm images (when compared to 850 nm) may very well be the reason for the slight inaccuracies (especially in deeper features) in our sO ${}_{2}$ estimates [49]. Our future work will include the design and development of new LED arrays with optimal wavelengths and improved optical energy.

Even though we achieved promising results in this proof-of-concept study, there is a lot of scope in improving the quantitative nature of PA sO ${}_{2}$ imaging. In our proposed fluence compensation model, we segmented the phantom and tissue as two layers (water and soft tissue layer in phantom, water and muscle in mouse imaging, and water and soft tissue in human wrist imaging), which is not an optimal approach. In our future work, we consider using the US images to segment different tissue types [50] and then perform a more accurate fluence compensation for quantitative sO ${}_{2}$ imaging. In the Monte-Carlo light propagation model, the reflection of light at different tissue layers was not considered, which will be considered in our future work. Another factor to consider in future studies is the band-limited frequency response of the detectors and its impact on the accuracy of sO ${}_{2}$ imaging. PA sO ${}_{2}$ is limited in predicting the absolute sO ${}_{2}$ values, as imaging depends on multiple factors, and it is difficult to calibrate the method in live tissue. However, relative sO ${}_{2}$ imaging is still useful in clinical applications. We foresee several applications including monitoring hypoxia in the tumor microenvironment in the future.

In summary, for the first time, we characterized and validated fluence-compensated LED-PAI based sO ${}_{2}$ imaging using in vitro, phantom and in vivo small animal and human volunteer studies. Our results show the importance of accounting for fluence variations in tissue when performing sO ${}_{2}$ using an LED-PAI system. Further, our results demonstrate the potential of LED-PAI in obtaining sO ${}_{2}$ information dynamically along with conventional pulse-echo imaging. We believe that combined US and sO ${}_{2}$ information in such high temporal and spatial resolution may be useful for diagnosis and treatment monitoring of several inflammatory and cancer-related diseases.

## 5. Conclusions

Through our results, we have demonstrated that oxygen saturation imaging using a dual-wavelength LED-based photoacoustic system has potential in pre-clinical and clinical applications. The proposed fluence compensation method utilizing structural information of the tissue from the US images was found to improve the oxygen saturation imaging accuracy by 12–15%. Within the limited power of the LEDs, an imaging depth of 6–8 mm was achieved in soft tissue. We have demonstrated imaging different levels of oxygenated blood in vivo in both small animal and a healthy human subject and found it to be within the expected range, showing the accuracy of the proposed approach. Further research on improving the pulse energy of LED sources to enhance imaging depth and accurate segmentation of different tissue types for fluence compensation can potentially translate this technology for pre-clinical and clinical applications.

## Figures and Tables

**Table 1:** Optical properties used in the simulations, optical absorption coefficient ( $\mu_{a}$ ) and reduced scattering coefficient ( $\mu_{s}^{\prime }$ ) (anisotropy, g = 0.9 ).

Medium	Wavelength [nm]	$\mu_{a}$ [cm ${}^{-1}$ ]	$\mu_{s}^{\prime }$ [cm ${}^{-1}$ ]
Soft tissue phantom [42]	750	0.101	10.5
	850	0.089	9
Mice thigh muscle [43]	850	0.76	5.3
	850	0.64	4.8
Human forearm [44]	750	0.41	7.2
	850	0.3	6.5

## Data Availability

Data available on request from the authors.

## Supplementary Materials

The following are available online at https://www.mdpi.com/1424-8220/21/1/283/s1, Video S1: Real-time ultrasound and photoacoustic oxygen saturation imaging of a human wrist, showing a pulsating artery, vein, and skin layer.

## References

[1] Vaupel P., Kallinowski F., Okunieff P. (1989). Blood flow, oxygen and nutrient supply, and metabolic microenvironment of human tumors: A review. Cancer Res..

[2] Cairns R.A., Harris I.S., Mak T.W. (2011). Regulation of cancer cell metabolism. Nat. Rev. Cancer.

[3] Mallidi S., Luke G.P., Emelianov S. (2011). Photoacoustic imaging in cancer detection, diagnosis, and treatment guidance. Trends Biotechnol..

[4] Szekanecz Z., Koch A.E. (2007). Mechanisms of disease: Angiogenesis in inflammatory diseases. Nat. Clin. Pract. Rheumatol..

[5] Diot G., Metz S., Noske A., Liapis E., Schroeder B., Ovsepian S.V., Meier R., Rummeny E., Ntziachristos V. (2017). Multispectral optoacoustic tomography (MSOT) of human breast cancer. Clin. Cancer Res..

[6] Petrova I., Petrov Y., Esenaliev R., Deyo D., Cicenaite I., Prough D. (2009). Noninvasive monitoring of cerebral blood oxygenation in ovine superior sagittal sinus with novel multi-wavelength optoacoustic system. Opt. Express.

[7] Zauner A., Daugherty W.P., Bullock M.R., Warner D.S. (2002). Brain oxygenation and energy metabolism: Part I—biological function and pathophysiology. Neurosurgery.

[8] Tang J., Coleman J.E., Dai X., Jiang H. (2016). Wearable 3-D photoacoustic tomography for functional brain imaging in behaving rats. Sci. Rep..

[9] Dale A.M., Halgren E. (2001). Spatiotemporal mapping of brain activity by integration of multiple imaging modalities. Curr. Opin. Neurobiol..

[10] Halmos G.B., de Bruin L.B., Langendijk J.A., van der Laan B.F., Pruim J., Steenbakkers R.J. (2014). Head and neck tumor hypoxia imaging by 18F-fluoroazomycin-arabinoside (18F-FAZA)-PET: A review. Clin. Nucl. Med..

[11] Wang L.V. (2008). Prospects of photoacoustic tomography. Med. Phys..

[12] Xu M., Wang L.V. (2006). Photoacoustic imaging in biomedicine. Rev. Sci. Instrum..

[13] Attia A.B.E., Balasundaram G., Moothanchery M., Dinish U., Bi R., Ntziachristos V., Olivo M. (2019). A review of clinical photoacoustic imaging: Current and future trends. Photoacoustics.

[14] Singh M.K.A., Steenbergen W., Manohar S. (2016). Handheld probe-based dual mode ultrasound/photoacoustics for biomedical imaging. Frontiers in Biophotonics for Translational Medicine.

[15] Vogt W.C., Zhou X., Andriani R., Wear K.A., Pfefer T.J., Garra B.S. (2019). Photoacoustic oximetry imaging performance evaluation using dynamic blood flow phantoms with tunable oxygen saturation. Biomed. Opt. Express.

[16] Li M., Tang Y., Yao J. (2018). Photoacoustic tomography of blood oxygenation: A mini review. Photoacoustics.

[17] Cox B.T., Laufer J.G., Beard P.C., Arridge S.R. (2012). Quantitative spectroscopic photoacoustic imaging: A review. J. Biomed. Opt..

[18] Manohar S., Razansky D. (2016). Photoacoustics: A historical review. Adv. Opt. Photonics.

[19] Kuniyil Ajith Singh M., Xia W. (2020). Portable and Affordable Light Source-Based Photoacoustic Tomography. Sensors.

[20] Upputuri P.K., Pramanik M. (2018). Fast photoacoustic imaging systems using pulsed laser diodes: A review. Biomed. Eng. Lett..

[21] Allen T.J. (2020). High-Power Light Emitting Diodes; An Alternative Excitation Source for Photoacoustic Tomography. LED-Based Photoacoustic Imaging.

[22] Sato N., Singh M.K.A., Shigeta Y., Hanaoka T., Agano T. (2018). High-speed photoacoustic imaging using an LED-based photoacoustic imaging system. Photons Plus Ultrasound: Imaging and Sensing 2018.

[23] Zhu Y., Feng T., Cheng Q., Wang X., Du S., Sato N., Yuan J., Kuniyil Ajith Singh M. (2020). Towards Clinical Translation of LED-Based Photoacoustic Imaging: A Review. Sensors.

[24] Mackle E., Maneas E., Xia W., West S., Desjardins A. (2020). LED-Based Photoacoustic Imaging for Guiding Peripheral Minimally Invasive Procedures. LED-Based Photoacoustic Imaging.

[25] Zhu Y., Xu G., Yuan J., Jo J., Gandikota G., Demirci H., Agano T., Sato N., Shigeta Y., Wang X. (2018). Light emitting diodes based photoacoustic imaging and potential clinical applications. Sci. Rep..

[26] Xavierselvan M., Singh M.K.A., Mallidi S. (2020). In Vivo Tumor Vascular Imaging with Light Emitting Diode-Based Photoacoustic Imaging System. Sensors.

[27] Singh M.K.A., Agano T., Sato N., Shigeta Y., Uemura T. (2018). Real-time in vivo imaging of human lymphatic system using an LED-based photoacoustic/ultrasound imaging system. Photons Plus Ultrasound: Imaging and Sensing 2018.

[28] Hariri A., Chen F., Moore C., Jokerst J.V. (2019). Noninvasive staging of pressure ulcers using photoacoustic imaging. Wound Repair Regen..

[29] Joseph F.K., Xavierselvan M., Singh M.K.A., Mallidi S., Van Der Laken C., Van De Loo F., Steenbergen W. (2020). LED-based photoacoustic imaging for early detection of joint inflammation in rodents: Towards achieving 3Rs in rheumatoid arthritis research. Photons Plus Ultrasound: Imaging and Sensing 2020.

[30] Francis K.J., Booijink R., Bansal R., Steenbergen W. (2020). Tomographic Ultrasound and LED-Based Photoacoustic System for Preclinical Imaging. Sensors.

[31] Francis K.J., Boink Y.E., Dantuma M., Singh M.K.A., Manohar S., Steenbergen W. (2020). Tomographic imaging with an ultrasound and LED-based photoacoustic system. Biomed. Opt. Express.

[32] Guo Z., Hu S., Wang L.V. (2010). Calibration-free absolute quantification of optical absorption coefficients using acoustic spectra in 3D photoacoustic microscopy of biological tissue. Opt. Lett..

[33] Kirchner T., Gröhl J., Maier-Hein L. (2018). Context encoding enables machine learning-based quantitative photoacoustics. J. Biomed. Opt..

[34] Bench C., Hauptmann A., Cox B.T. (2020). Toward accurate quantitative photoacoustic imaging: Learning vascular blood oxygen saturation in three dimensions. J. Biomed. Opt..

[35] Tzoumas S., Nunes A., Olefir I., Stangl S., Symvoulidis P., Glasl S., Bayer C., Multhoff G., Ntziachristos V. (2016). Eigenspectra optoacoustic tomography achieves quantitative blood oxygenation imaging deep in tissues. Nat. Commun..

[36] Hussain A., Petersen W., Staley J., Hondebrink E., Steenbergen W. (2016). Quantitative blood oxygen saturation imaging using combined photoacoustics and acousto-optics. Opt. Lett..

[37] Kim S., Chen Y.S., Luke G.P., Emelianov S.Y. (2011). In vivo three-dimensional spectroscopic photoacoustic imaging for monitoring nanoparticle delivery. Biomed. Opt. Express.

[38] Singh M.K.A., Sato N., Ichihashi F., Sankai Y. (2019). In vivo demonstration of real-time oxygen saturation imaging using a portable and affordable LED-based multispectral photoacoustic and ultrasound imaging system. Photons Plus Ultrasound: Imaging and Sensing 2019.

[39] Li M.L., Oh J.T., Xie X., Ku G., Wang W., Li C., Lungu G., Stoica G., Wang L.V. (2008). Simultaneous molecular and hypoxia imaging of brain tumors in vivo using spectroscopic photoacoustic tomography. Proc. IEEE.

[40] Jaeger M., Schüpbach S., Gertsch A., Kitz M., Frenz M. (2007). Fourier reconstruction in optoacoustic imaging using truncated regularized inverse k-space interpolation. Inverse Probl..

[41] Fang Q., Boas D.A. (2009). Monte Carlo simulation of photon migration in 3D turbid media accelerated by graphics processing units. Opt. Express.

[42] Jacques S.L. (2013). Optical properties of biological tissues: A review. Phys. Med. Biol..

[43] Krainov A., Mokeeva A., Sergeeva E., Agrba P., Kirillin M.Y. (2013). Optical properties of mouse biotissues and their optical phantoms. Opt. Spectrosc..

[44] Bashkatov A.N., Genina E.A., Tuchin V.V. (2011). Optical properties of skin, subcutaneous, and muscle tissues: A review. J. Innov. Opt. Health Sci..

[45] Allen T.J., Beard P.C. (2016). High power visible light emitting diodes as pulsed excitation sources for biomedical photoacoustics. Biomed. Opt. Express.

[46] Hauptmann A., Cox B. (2020). Deep Learning in Photoacoustic Tomography: Current approaches and future directions. J. Biomed. Opt..

[47] Anas E.M.A., Zhang H.K., Kang J., Boctor E. (2018). Enabling fast and high quality LED photoacoustic imaging: A recurrent neural networks based approach. Biomed. Opt. Express.

[48] Farnia P., Najafzadeh E., Hariri A., Lavasani S.N., Makkiabadi B., Ahmadian A., Jokerst J.V. (2020). Dictionary learning technique enhances signal in LED-based photoacoustic imaging. Biomed. Opt. Express.

[49] Hochuli R., An L., Beard P.C., Cox B.T. (2019). Estimating blood oxygenation from photoacoustic images: Can a simple linear spectroscopic inversion ever work?. J. Biomed. Opt..

[50] Han T., Yang M., Yang F., Zhao L., Jiang Y., Li C. (2020). A three-dimensional modeling method for quantitative photoacoustic breast imaging with handheld probe. Photoacoustics.

