# Correction: Bulsink et al. Oxygen Saturation Imaging Using LED-Based Photoacoustic System. *Sensors* 2021, *21*, 283

**DOI:** 10.3390/s22134839

**Published:** 2022-06-27

**Authors:** Rianne Bulsink, Mithun Kuniyil Ajith Singh, Marvin Xavierselvan, Srivalleesha Mallidi, Wiendelt Steenbergen, Kalloor Joseph Francis

**Affiliations:** 1 Biomedical Photonic Imaging (BMPI), Technical Medical Center, University of Twente, 7500 AE Enschede, The Netherlands; r.e.bulsink@student.utwente.nl (R.B.); w.steenbergen@utwente.nl (W.S.); 2 Research & Business Development Division, CYBERDYNE Inc., Cambridge Innovation Center, 3013 AK Rotterdam, The Netherlands; mithun_ajith@cyberdyne.jp; 3 Department of Biomedical Engineering, Science and Technology Center, Tufts University, Medford, MA 02155, USA; marvin.xavierselvan@tufts.edu (M.X.); srivalleesha.mallidi@tufts.edu (S.M.); 4 Wellman Center for Photomedicine, Massachusetts General Hospital, Harvard Medical School, Boston, MA 02114, USA

The authors wish to make the following corrections to this paper [1]:

## 1. Change in Figure 1

The authors wish to correct the text in Figure 1. In the published article, there was a mistake in the text of Figure 1f. The text “60%(Air)” in the published article is replaced by “Air”. The corrected figure appears below.

**Figure:**
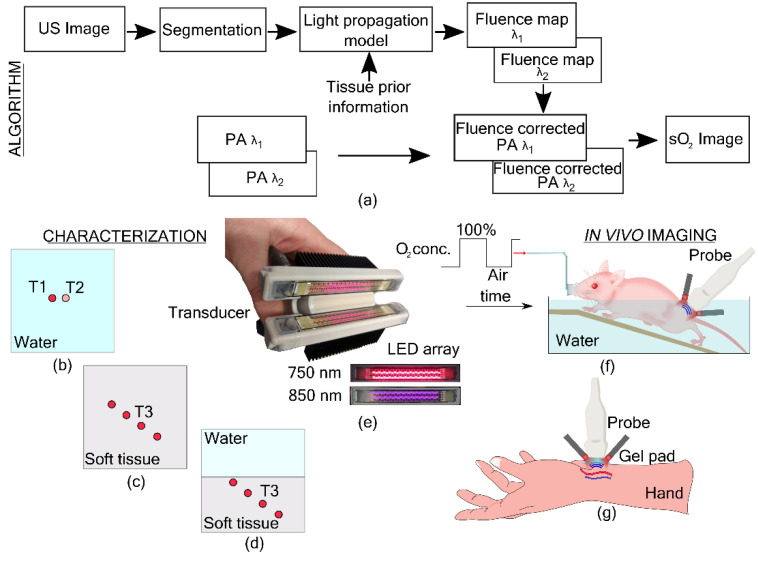


## 2. Change in Figure 4

The authors wish to make a correction to the text in Figure 4. The title of Figure 4g, “sO_2_ before fluence compensation” in the published article is replaced by “sO_2_ after fluence compensation”. The corrected figure appears below.

**Figure:**
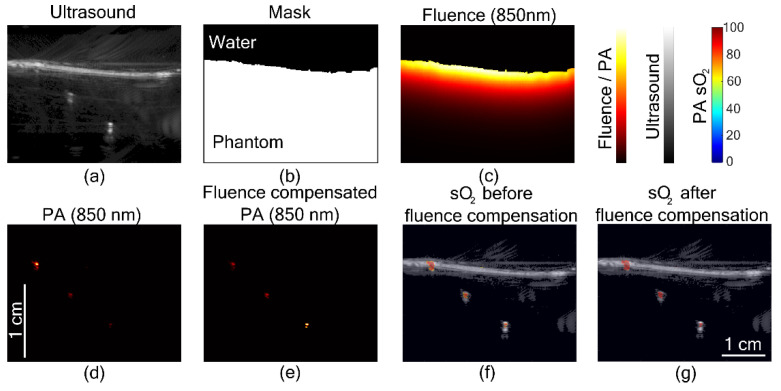


## 3. Change in Figure 6

The authors wish to correct the x-axis of Figure 6b. With dual-wavelength imaging, the frame rate becomes half, which was not considered in the original article by mistake. The corrected figure appears below.

**Figure:**
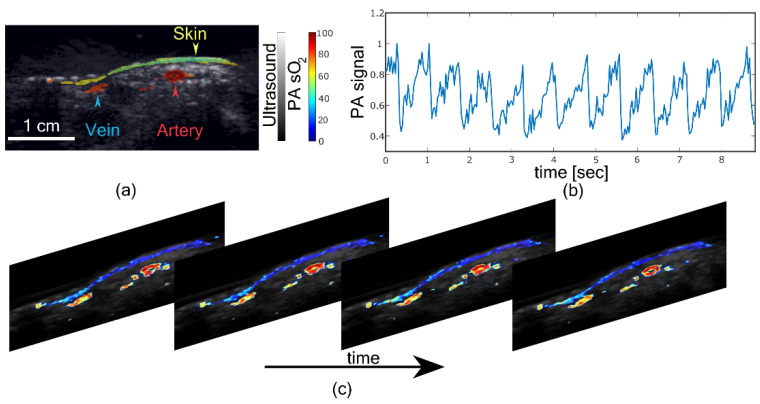


The authors apologize for any inconvenience caused and state that the scientific conclusions are unaffected. The original article has been updated.
